# Development of a new duplex real-time polymerase chain reaction assay for hepatitis B viral DNA detection

**DOI:** 10.1186/1743-422X-8-227

**Published:** 2011-05-14

**Authors:** Shipeng Sun, Shuang Meng, Rui Zhang, Kuo Zhang, Lunan Wang, Jinming Li

**Affiliations:** 1 National Center for Clinical Laboratories, Beijing Hospital, Beijing, People's Republic of China; 2 Graduate School, Peking Union Medical College, Chinese Academy of Medical, Sciences, Beijing, People's Republic of China; 3 State Key Laboratory for Infectious Disease Prevention and Control, National Institute for Communicable Disease Control and Prevention, Chinese Center for Disease Control and Prevention, Beijing, PR China

## Abstract

**Background:**

Quantification of hepatitis B virus (HBV) DNA can be used for diagnosing HBV infection and monitoring the effect of antiviral therapy. However, probably because of mismatches between the template and primer/probe, HBV DNA in some HBV infections could not be detected using currently available commercial assays with single primer/probe. By aligning the HBV sequences, we developed a duplex real-time polymerase chain reaction (PCR) assay using two sets of primers/probes and a specific armored DNA as internal control (IC).

**Results:**

The limit of the duplex real-time PCR assay was 29.5 IU/ml, whereas the specificity was 100%. The within-run precision coefficient of variation (CV) ranged from 1.02% to 2.73%, while the between-run CV ranged from 0.83% to 1.25%. The optimal concentration of armored DNA IC in the HBV DNA duplex real-time PCR assay was 1 000 copies/ml. Data from 69 serum samples with HBV infection showed that the performance of the duplex real-time PCR assay was comparable to that of the COBAS Ampliprep/Cobas Taqman (CAP/CTM) HBV assay and was superior to those of the domestic commercial HBV assays.

**Conclusions:**

The duplex real-time PCR assay is sufficiently sensitive, specific, accurate, reproducible and cost-effective for the detection of HBV DNA. It is suitable for high throughput screening and frequent HBV DNA level monitoring.

## Background

An estimated 600,000 persons worldwide die each year due to the acute or chronic consequences of hepatitis B caused by the hepatitis B virus (HBV) infection [1]. Nowadays, HBV infection is a leading cause of death in China [2]. Of the 350-400 millions people with chronic hepatitis B, a third of them live in China [3]. Serologic tests were used routinely for the diagnosis of HBV infection. However, during the window period of hepatitis B virus infection, early diagnosis and follow-up of infection cannot be achieved by serologic tests. Moreover, some studies indicate that HBV may be transmitted by individuals with occult HBV infection (OHB), that is, persons who have no serologic evidences of ongoing HBV replication [4,5].

The best indication of active viral replication is detection of HBV DNA in plasma or serum [6-9]. Several assays based on real-time PCR have been developed for quantification of HBV DNA in serum or plasma samples [10-12]. Compared to serologic tests, real-time PCR-based assays with high sensitivity and high specificity may allow earlier diagnosis of HBV infection [13,14]. HBV polymerase lacks proofreading activity, thus, the mutation rate for HBV is higher than the rate observed for most DNA viruses [15,16]. Currently there are eight accepted genotypes (A to H) for HBV based on the inter-group divergence of 8% or more in the complete genome sequence [17]. High mutation rate of viral genomes may results in failure to recognize increasing viremia levels [18] and even miss detecting HBV DNA by real-time PCR assay with single primer/probe because of mismatches between the template and the primer/probe [19,20]. Previous studies indicated that the performances of duplex real-time reverse transcriptase-PCR assay have been improved and could avoid missing detection of hepatitis C virus (HCV) and Human immunodeficiency virus (HIV) to some extent with two sets of primer/probe [21,22]. Similarly, a real-time PCR assay with two sets of primer/probe may resolve the problem of mismatches and avoid missing detections of HBV infection. However, rare information has been published on the duplex real-time PCR assay for HBV DNA quantification.

In addition, it is necessary to use an internal control (IC) to monitor the specimen extraction and amplification efficiency of real-time PCR assay. Compared with commonly used plasmid IC, armored DNA produced by the lambda phage system is DNase-resistant, stable, noninfectious, inexpensive, and easily to be extracted and could be used as an ideal control for clinical viral testing [23-25].

In this study we developed a duplex real-time PCR assay with armored DNA as IC for HBV DNA detection. The assay possesses all the performance characteristics that make it amenable for high throughput screening of HBV infection.

## Materials and methods

### Serum samples and standards

30 HBV-positive and 10 HBV-negative samples from Beijing Blood Center (Beijing, China) were used for comparison of the performances of singleplex primer/probe and duplex primer/probe assays.

100 HBV-negative blood donors serum samples including 80 healthy controls and 20 controls with hepatitis A, hepatitis C, hepatitis E, human immunodeficiency virus type 1 infection, or human T-cell leukaemia virus infection (confirmed at the blood bank) were enrolled.

Furthermore, 69 serum samples were collected from Shenzhen Blood Center (Guangdong, China). Each sample was divided into 4 aliquots and frozen at -80°C within 4 h after collection. These samples were used to compare the performances of Kehua HBV DNA real-time PCR detection kit (Shanghai Kehua Bio-Engineering Co. Ltd., Shanghai, China), qualitative duplex real-time PCR assay, and COBAS Ampliprep/Cobas Taqman (CAP/CTM) assay (Roche Molecular Systems, Pleasanton, CA).

A dilution series of the World Health Organization (WHO) Second International Standard for HBV DNA (National Institute for Biological Standards and Control (NIBSC), code 97/750, UK) was used to determine the limit of detection (LOD) of the duplex real-time PCR assay at the following concentrations: 5, 10, 25, 50, 10^2^, 10^3^, 10^4 ^and 10^5 ^IU/ml. Each dilution of the WHO Standard was tested in a batch of 4 replicates in 6 separate runs, i.e. for each dilution, a total of 24 replicates were tested.

### Selection of duplex real-time PCR primers and probes

In order to identify optimal sites for primers design, 44 HBV complete genome reference sequences representative of all eight genotypes (A-H) downloaded from GenBank Database were aligned using DNA star software. GeneBank accession numbers of 44 HBV complete genome reference sequences are genotypes A (AB194951, AF090842, AJ309369, AY217375, U87742, X02763, X51970); genotypes B (AB010292, AB031266, AB033554, AB033555, AF100309, D00329, U87747); genotypes C (AB014381, AB048704, AB049609, AB241110, AY123041, X01587, X04615, X75665); genotypes D (AY090453, M32138, V01460, X65259, X85254); genotypes E (AB032431, DQ060823, X75657); genotypes F (AB036905, AB036910, AB086397, AF223962, AF223965, AY090455, X69798); genotypes G (AB064310, AF160501, AF405706); genotypes G (AB059661, AY090454, AY090457, AY090460). Based on the consensus sequences of the HBV genome, two sets of primer/probe (A and B) targeting S genes and probe for IC (ICp) were designed (Table 1). To ensure the primers only amplify HBV DNA, they were tested by the BLAST algorithm.

**Table 1:** Primer and probe sequences

Primer or probe	Sequence (5'-3')	Nucleotide Position
Af	5'- GTCCTCCAATTTGTCCTGG -3'	2196-2214
Ar	5'- TGAGGCATAGCAGCAGGAT -3'	2276-2258
Ap	5' FAM - CTGGATGTGTCTGCGGCGTTTTATCAT - BHQ 3'	2221-2247
Bf	5'- CACCTGTATTCCCATCCCATC -3'	2443-2458
Br	5'- AGCCCTACGAACCACTGAACA -3'	2559-2539
Bp	5' FAM - AAACGGACTGAGGCCCACTCCCA -BHQ 3'	2511-2489
ICp	5' Cy5 - CCCCCCCCCCCCCCAAAAAAAA -BHQ 3'	

Nucleotide sequence positions were numbered according to reference sequence AF090842 (genotype A). Probes for the detection of HBV DNA and IC were labelled with 6-carboxyfluorescein (FAM) and cyanine dye (Cy5) at the 5' end, respectively. The 3' ends of probes were labelled with Black Hole Quencher dye (BHQ). The length of PCR products amplified from set A and B are 81 bp and 117 bp, respectively.

### Construction and selection of optimal concentration of IC

Armored DNA was produced with the same method as described [23]. The sequences of IC were identical to the wild-type HBV sequences, except for the sequences of probe Ap- and probe Bp-binding site, which were replaced by the internal probe sequences (5' CCCCCCCCCCCCCCAAAAAAAA 3'). A chequerboard assay was performed in which the international reference material for HBV DNA (NIBSC 97/746; 0 IU/ml, 5 × 10^2 ^IU/ml, 5 × 10^3 ^IU/ml, 5 × 10^4 ^IU/ml, 5 × 10^5 ^IU/ml) were spiked with four different copy numbers (10^5^, 10^4^, 10^3^, 0) of the armored DNA. Armored DNA was extracted and amplified with the international reference material for HBV DNA in the same reaction tube.

### Duplex real-time PCR and singleplex real-time PCR amplification for HBV DNA detection

DNA was extracted from 100 μl sample by using extraction reagents of the Kehua HBV DNA real-time PCR detection kit according to the manufacturer's instructions. 1.0 μl armored DNA particles (1000 copies/μl), added to each sample prior to extraction, were used as ICs of the extraction and amplification processes. The final optimized PCR mixture (25 μl) contained 12.5 μl QuantiTect Probe PCR Master Mix (QIAGEN, QuantiTect Multiplex PCR kit), 1.0 μl armored DNA particles, 8 μl HBV DNA samples and the concentration of primer/probe were added as follows, in the singleplex mode, either the primer/probe set A (0.4 μM primers, 0.4 μM probes, and 0.2 μM IC-specific probe) or the primer/probe set B (0.4 μM primers, 0.4 μM probes, and 0.2 μM IC-specific probe), was used in the reaction, whereas in the duplex mode, both the primer/probe sets A (0.4 μM primers, 0.4 μM probes, and 0.2 μM IC-specific probe) and B (0.4 μM primers, 0.4 μM probes, and 0.2 μM IC-specific probe) were used in PCR (0.4 μM primers, 0.4 μM probes, and 0.2 μM IC-specific probe). PCR was performed with an ABI 7500 sequence detection system as follows: an initial denaturation step at 95°C for 15 min, 45 cycles at 94°C for 15 s and 60°C for 1 min.

### Evaluation of the duplex real-time PCR assay

To establish the linearity of HBV DNA quantification, we prepared serial 10-fold dilutions from the HBV standard in negative serum to obtain concentrations of 10, 10^2^, 10^3^, 10^4^, 10^5^, and 10^6 ^IU/ml. Each concentration was tested three replicates in a single run.

A set of 3 HBV positive serum samples with different viral loads was tested 10 times in a single run to determine within-run precision coefficient of variation (CV) in the viral quantification. Similarly, the same set of samples was quantified in 10 different experiments to determine between-run CV in the viral quantification. The coefficients of variance (CV) of the threshold cycles (Ct) were calculated.

### Commercial kits for HBV DNA detection

A total of 69 serum samples were tested using Kehua HBV DNA real-time PCR detection kit and CAP/CTM assay kit. All the operation steps were carried out according to the instructions given in the manuals.

(i) Detection using Kehua HBV DNA real-time PCR detection kit. HBV DNA was extracted from 100 μl sample, and 12.5 μl extract was used as the template in 25 μl reaction. PCR was carried out in a 32-well Lightcycler thermal cycles system (Roche). HBV DNA levels were expressed in IU/ml. The LOD of Kehua HBV DNA assay kit was 500 IU/ml.

(ii) Detection using CAP/CTM HBV assay kit. The CAP/CTM test utilized automated specimen preparation on the COBAS AmpliPrep Instrument by a generic silica-based capture technique. HBV DNA was extracted from 850 μl serum and then eluted with 65 μl of elution buffer. Finally, 50 μl extract was used as the template in 100 μl reaction volume. The COBAS TaqMan 48 Analyzer was used for automated real-time PCR amplification. HBV DNA levels were expressed in IU/ml. The LOD of CAP/CTM HBV assay kit was 12 IU/ml.

### Comparison the duplex primer/probe real-time PCR assays with the singleplex primer/probe real-time PCR assays for HBV DNA detection

The results of 30 HBV-positive and 10 HBV-negative samples tested by singleplex primer/probe and duplex primer/probe assays were compared.

### Comparison the duplex primer/probe real-time PCR assay with commercial kits for HBV DNA detection

69 serum samples were tested by Kehua HBV DNA real-time PCR detection kit, qualitative duplex real-time PCR assay, and COBAS CAP/CTM assay, the results were then compared.

### Data analysis

Results were expressed as means and standard deviation (SD). The intra-assay and inter-assay variations were expressed as coefficient of variation (CV), based on the mean Ct values. Probit analysis was performed to determine the LOD. The LOD was determined as 95% probability of obtaining a positive HBV DNA result. Correlation coefficients (R) were calculated for linearity data.

## Results

### Optimal concentration of IC

To establish the optimum copy number of internal control to be added to the tubes for HBV DNA duplex real-time PCR assay, a chequerboard assay was performed in which serially diluted HBV standards (5 × 10^5 ^to 0 IU/ml) were spiked with four different copy numbers (10^5 ^to 0) of the internal control armored DNA (Table 2). The IC copies more than 1 000 changed the threshold cycles (Ct) for almost all the standards which resulted in the underestimation of the copy number. The optimal concentration of armored DNA IC in the HBV DNA duplex real-time PCR assay was 1 000 copies/ml.

**Table 2:** Optimization of the concentration of IC

Armored DNA concentration (copies/ml)	National reference material 4 for HBV DNA 5 × 10^5 ^IU/ml		National reference material 3 for HBV DNA 5 × 10^4 ^IU/ml		National reference material 2 for HBV DNA 5 × 10^3 ^IU/ml		National reference material 1 for HBV DNA 5 × 10^2^IU/ml		HBV 0 IU/ml
	
	IC (Cy5)Ct	HBV (FAM)Ct	IC (Cy5)Ct	HBV (FAM)Ct	IC (Cy5)Ct	HBV (FAM)Ct	IC (Cy5)Ct	HBV (FAM)Ct	IC (Cy5)Ct
100 000	38.92	28.00	34.43	32.17	31.18	35.78	30.33	> 45	30.13
10 000	41.51	28.93	38.39	31.20	35.58	34.29	34.29	39.55	34.21
1 000	> 45	23.14	38.35	26.44	37.72	29.23	35.68	32.67	35.47
0	> 45	23.21	> 45	26.11	> 45	29.14	> 4 5	32.82	> 45

Concentrations of HBV DNA and armored DNA were indicated by FAM and Cy5 signals, respectively.

### Evaluation of the duplex real-time PCR assay

**Figure 1:**
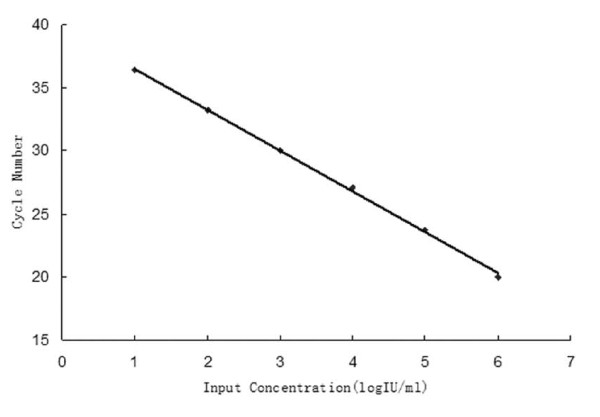
**Linearity of the duplex real-time PCR assay**. Linearity of the duplex real-time PCR assay was determined using serial 10-fold dilutions of HBV standard in negative serum samples at the following concentrations: 10, 10^2^, 10^3^, 10^4^, 10^5^, and 10^6 ^IU/ml. Three replicates were tested in a single run at each concentration. Linear relationship between the Ct values and the log10 HBV DNA concentration in the samples at concentrations from 10 to 106 IU/ml yielded R = 0.993.

Linearity of the duplex real-time PCR assay was determined using serial 10-fold dilutions of HBV standard in negative serum samples at the following concentrations: 10, 10^2^, 10^3^, 10^4^, 10^5^, and 10^6 ^IU/ml. Three replicates were tested in a single run at each concentration. The proportion of positive results obtained from each input concentration was subjected to probit regression analysis (Table 3). The LOD of the duplex real-time PCR assay was 29.5 IU/ml (95% confidence interval, 20.9-56.2 IU/ml). Linear regression analysis of the Ct values against the log10 HBV DNA concentration yielded R = 0.993 (Figure 1). The specificity of the duplex real-time PCR assay was 100% when testing HBV-negative serum samples.

**Table 3:** Limit of detection of the duplex real-time PCR assay

HBV load (IU/ml)	Positive results/total tested	Positive results (%)
10^5^	24/24	100
10^4^	24/24	100
10^3^	24/24	100
10^2^	24/24	100
50	24/24	100
25	22/24	91.6
10	12/24	50.0
5	5/24	20.8

The intra-assay variation was assessed by testing 3 samples with different viral loads (10^5^, 10^4^, and 10^2 ^IU/ml) 10 times in a single run, while the inter-assay variation was assessed by testing the same samples 10 times in 10 separate runs. The intra-assay CV ranged from 0.83% to 1.25%, while the inter-assay CV ranged from 1.02% to 2.73% (Table 4).

**Table 4:** Reproducibility of the duplex real-time PCR assay

Reproducibility	Target HBV RNA (IU/ml)	Number of determinations	Mean Ct	SD	CV (%)
Intra-assay	10^5^	10	24.15	0.24	0.83
	10^4^	10	27.09	0.34	1.07
	10^2^	10	33.31	0.49	1.25
Inter-assay	10^5^	10	24.05	0.27	1.02
	10^4^	10	27.05	0.23	0.77
	10^2^	10	33.23	1.02	2.73

### Comparison the duplex primer/probe real-time PCR assays with the singleplex primer/probe real-time PCR assays for HBV DNA detection

**Figure 2:**
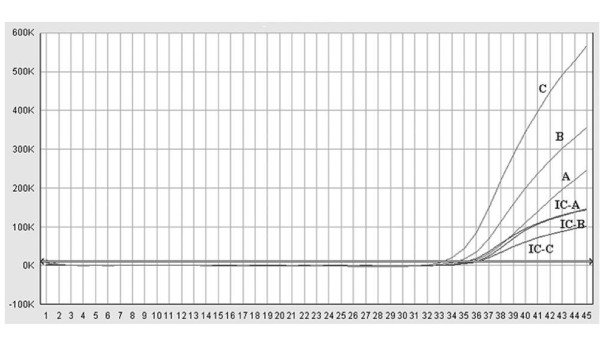
**Comparison the duplex primer/probe real-time PCR assays with the singleplex primer/probe real-time PCR assays for HBV DNA detection**. The performances of the duplex primer/probe (C) and singleplex primer/probe (A, B) assays in the testing of the same serum sample obtained from a patient with low HBV viraemia were compared. The red amplification curves represent FAM fluorescence signal and the blue amplification curves represent Cy5 fluorescence signal. A: amplification plot of the HBV sample in the singleplex primer/probe A reaction system. B: amplification plot of the HBV sample in the singleplex primer/probe B reaction system. C: amplification plot of the HBV sample in the duplex primer/probe A and B reaction system. The Cy5 fluorescence signals indicate the amplification of IC. IC-A represents the amplification plot of ICs used in the singleplex primer/probe A reaction system. IC-B represents the amplification plot of ICs used in the singleplex primer/probe B reaction system. IC-C represents the amplification plot of ICs used in the duplex primer/probe A and B reaction system.

40 serum samples were submitted for routine HBV DNA testing. The duplex primer/probe assay could strengthen the fluorescence signal of the low HBV viraemia samples. Figure 2 showed the performance of the duplex primer/probe (C) and singleplex primer/probe (A, B) assays in the testing the same low-load HBV sample. Of the confirmed 30 HBV DNA positive samples, both singleplex primer/probe set A and set B failed to detect 2 low HBV viraemia samples. In contrast, the duplex primer/probe sets A+B detected all the 30 HBV-positive serum samples (Table 5).

**Table 5:** Testing results of duplex real-time PCR assay and singleplex real-time PCR assay for 40 serum samples

Duplex primer/probe assay	Singleplex primer/probe assay (set A primer/probe)	Singleplex primer/probe assay (set B primer/probe)	Number of samples detected
+	+	+	28
+	-	-	2
-	-	-	10

+: positive result

-: negative result

### Comparison the duplex primer/probe real-time PCR assay with commercial kits for HBV DNA detection

A set of 69 serum samples were tested in duplicate to verify the validity of the duplex primer/probe real-time PCR assay. Results obtained by the duplex real-time PCR assay were comparable to those obtained by the CAP/CTM assay. Kehua HBV DNA real-time PCR assay kit failed to detect 10 samples, which could be detected by the duplex primer/probe real-time PCR assay and the CAP/CTM assay (Table 6).

**Table 6:** Testing results of different assays and kits for 69 serum samples

Kehua HBV DNA real-time PCR detection kit	CAP/CTM HBV assay	Duplex primer/probe assay	Number of samples detected
+	+	+	45
-	+	+	10
-	-	-	14

+: positive result

-: negative result

## Discussion

In this study, we developed a novel HBV DNA quantification system using a real-time PCR assay which consisted of two sets of primer/probe and armored DNA as internal control in a single reaction tube. The primers and two FAM labelled probes were designed corresponding to the highly conserved HBV S gene. Compared with the singleplex primer/probe real-time PCR assay, the performance of duplex real-time PCR assay was remarkably improved and could avoid missing detection of HBV DNA to maximum extent with two sets of primer/probe.

The efficacy and accuracy of real-time PCR largely depend on the primers and probe [26]. It has been reported that several commercial HCV assays using a singleplex primer/probe set produce false-negative results because of mismatches between the template and primers/probes [27,28]. Similarly, a single primer/probe may result in failure to recognize increasing viremia levels [18] and even miss detections because of mismatches for HBV DNA detection. Theoretically, the assay developed in this study can detect all eight genotypes of HBV with the two sets of primer/probe, especially the genotype A, B, C and G of HBV which genome DNA can be matched by both sets of primer/probe. Data from 69 serum samples from patients with HBV infection showed that the duplex primer/probe assay developed for HBV DNA detection can avoid mismatches between primer/probe and virus DNA template to maximum extent. Of the confirmed 30 HBV DNA positive samples, two HBV-samples failed to detect by singleplex primer/probe set, but detected by duplex primer/probe set. The 2 sets of primers/probes used in our assay could match interchangeably, creating additional combinations with different primer-directed elongations. Figure 2 shows the performances of the duplex primer/probe and singleplex primer/probe assays in the testing of the same serum sample. Obviously, the fluorescence value of the 2 sets of primers/probes is higher than that of the single set of primers/probes, and cycle threshold (Ct) can shift towards left. As a result, the duplex primer/probe assay could strengthen the fluorescence signal of the low HBV viraemia samples and increase the probability of detection (Figure 2). The manufacturer's lower LOD of the CAP/CTM assay and Kehua HBV fluorescence detection kit are 12 IU/ml and 500 IU/ml, respectively. The sensitivity and the specificity of the duplex assay were comparable with the CAP/CTM assay and were superior to domestic Kehua HBV fluorescence detection kit. Furthermore, less serum samples (100 μl serum) and lower cost were required than the CAP/CTM assay.

In order to identify PCR-inhibited samples, an armored DNA was constructed as IC in PCR assays by means of the overlapping extension PCR technique. Unlike the plasmid-derived control which can only monitor the amplification process, armored DNA was co-extracted and co-amplified with the samples in the same reaction tube that can monitor the entire real-time PCR assay [23]. IC sequences were identical to the wild-type HBV sequences, except that the probe binding site sequences were replaced by the internal probe ones. The 5' ends of probes for the detection of HBV were labelled with 6-carboxyfluorescein (FAM), while probe for the detection of IC was labelled with cyanine dye (Cy5), so that the amplification of the IC could be distinguished easily from that of the HBV virus by different fluorophore. In order to avoid its suppression to target amplification, the concentration of competitive IC (CIC) spiked into the samples was optimized at 1000 copies/ml in the real-time assay (Table 2). Although with HBV standards number at 5 × 10^5 ^IU/ml, the amplification of the IC DNA (1000 copies/ml, Ct > 45) was competitively inhibited. It does not affect the inclusion of an internal control to monitor for false negative results due to DNA degradation or to inhibitory factors potentially present in clinical samples. By using this armored DNA as IC, false negative samples that resulted from inappropriate reagent additions in the tubes or the presence of PCR inhibitors can be easily identified. Here, no inhibitors were found in the clinical samples tested in the duplex primer/probe assay.

## Conclusions

We proved that two sets of primer/probe in the real-time PCR assay can effectively resolve the problem of mismatches and avoid missing detections of HBV infection. The established duplex real-time PCR assay is sufficiently sensitive, specific, accurate, reproducible and cost-effective for the detection of HBV DNA. It is suitable for high throughput screening and frequent HBV DNA level monitoring. Nevertheless, this study was carried out in China with a relatively small cohort, application of the duplex real-time PCR assay might be validated by larger serum samples including OHB serum samples.

## Competing interests

The authors declare that they have no competing interests.

## Authors' contributions

SPS and SM performed the experiments, participated in the sequence alignment and analyzed the data and drafted the manuscript. RZ, KZ and LNW involved in analyzing the data and revising the manuscript. JML conceived and designed the experiments, contributed reagents/materials/analysis tools. All authors read and approved the final manuscript.
